# YM155, a survivin suppressant, triggers PARP-dependent cell death (parthanatos) and inhibits esophageal squamous-cell carcinoma xenografts in mice

**DOI:** 10.18632/oncotarget.4315

**Published:** 2015-06-15

**Authors:** Nan Zhao, Yousheng Mao, Gaijing Han, Qiang Ju, Lanping Zhou, Fang Liu, Yang Xu, Xiaohang Zhao

**Affiliations:** ^1^ State Key Laboratory of Molecular Oncology, Cancer Institute & Hospital, Chinese Academy of Medical Sciences & Peking Union Medical College, Beijing, China; ^2^ Department of Thoracic Surgical Oncology, Cancer Hospital, Chinese Academy of Medical Sciences & Peking Union Medical College, Beijing, China

**Keywords:** PARP, parthanatos, chemotherapy, esophageal cancer

## Abstract

Here we demonstrated that sepantronium bromide (YM155), a survivin suppressant, inhibited esophageal squamous-cell carcinoma (ESCC) growth in mice bearing human ESCC xenografts without affecting body weight. In cell culture, YM155 decreased survivin levels and caused PARP-1 activation, poly-ADP polymer formation, and AIF translocation from the cytosol to the nucleus. Genetic knockdown of PARP-1 or AIF abrogated YM155-induced parthanatos cell death. Furthermore, FOS, JUN and c-MYC gene transcription, which is stimulated by activated PARP-1, was increased following YM155 treatment. Our data demonstrate that YM155 did not trigger apoptosis, but induced parthanatos, a cell death dependent on PARP-1 hyper-activation, and support clinical development of YM155 in ESCC.

## INTRODUCTION

Survivin is overexpressed in most human cancers, including lung, breast, pancreatic, and esophageal cancers [1–4]. YM155, a small-molecule transcriptional repressor of survivin, selected through high-throughput screening with a luciferase assay using a survivin promoter, is currently in phase II clinical trials [5]. Several malignant cancers, including non-small cell lung cancer, metastatic breast cancer, melanoma, and non-Hodgkin's lymphoma, were clinically evaluated with YM155 alone and as part of combination treatments (http://www.clinicaltrials.gov). YM155 alone or in combination decreases tumor growth, induces apoptosis, sensitizes resistant cells to apoptosis, and prolongs survival of tumor-bearing mice [6–8]. Previous data have demonstrated that YM155 mediates survivin suppression via directly binding and disrupting the interleukin enhancer-binding factor 3/NF110 transcription factor complex [9]. Recently, using a genome-wide insertional mutagenesis approach in the near-haploid human cell line KBM7, Kasap and colleagues demonstrated that YM155 is a general DNA intercalator that causes cytotoxicity via DNA damage and is dependent on the expression of the solute carrier gene SLC35F2[10]. There are additional factors affecting the cellular uptake of YM155, such as the human organic cation transporter 1 (OCT1/SLC22A1) in primary human hepatocytes.

Esophageal squamous-cell carcinoma (ESCC) ranks as the fourth most common cause of cancer-related death in China, with approximately 50% of total ESCC cases in the world occur in China [11, 12]. Most ESCC patients in China were diagnosed with advanced stages of the disease, which respond poorly to chemotherapy. Concurrent chemo/radiotherapy is currently the standard of care in the nonsurgical management of advanced esophageal cancer [13]. Cisplatin (cis-Diaminedichloroplatinum (II), CDDP), combined with 5-fluorouracil (5-FU), is a standard regimen that has been commonly used to treat ESCC clinically. A recent genome-wide association study (GWAS) searching for genetic variants associated with length of survival with ESCC identified the upregulation of a member of the 39A family of solute carrier proteins (SLC39A6) [14].

Here, we examined the expression of survivin and SLC35F2, responsible for YM155 uptake, in multiple esophageal cell lines, testing the antitumor activity of relevant YM155 concentrations against ESCC both *in vitro* and *in vivo*. In esophageal cancer cells, YM155 caused non-apoptotic cell death, a PARP-mediated cell death (parthanatos). We also investigated the molecular mechanism of PARP-mediated parthanatos. YM155 killed esophageal cancer cells by causing DNA damage and the hyper-activation of PARP-1 and the translocation of AIF, leading to PARP-1-dependent cell death. Genetic knockdown of PARP-1 or AIF abrogated YM155-induced parthanatos cell death, suggesting that PARP-1 and AIF were indispensable for YM155-mediated parthanatos induction. YM155 also reduced esophageal tumor growth and tumor weight in xenograft animal models without affecting mouse body weight. These data emphasize the significance of PARP-dependent parthanatos induction by YM155 and should encourage clinical trials in ESCC.

## RESULTS

### YM155 inhibits growth of esophageal cancer cell lines

**Figure 1:**
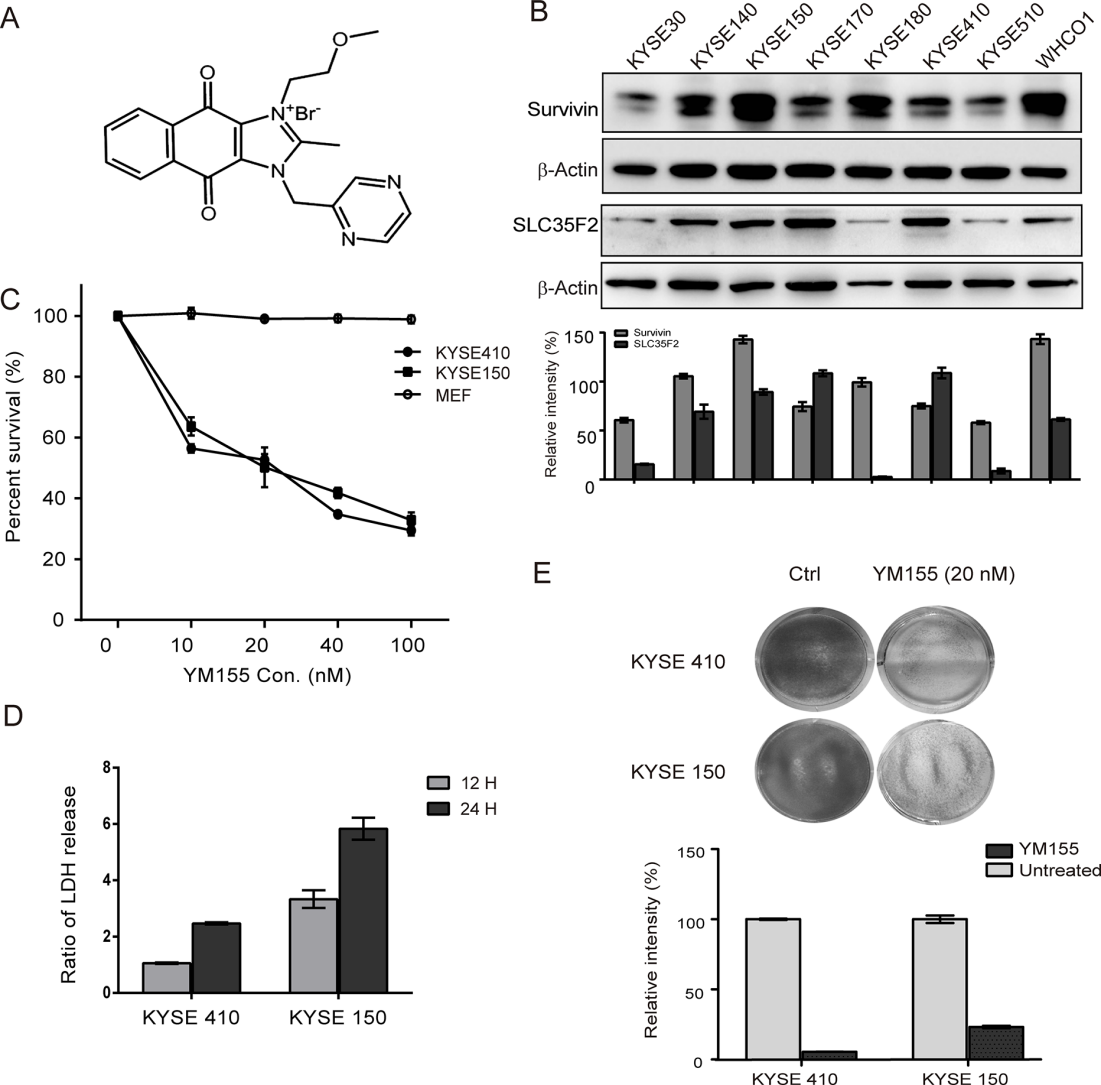
Effect of YM155 on esophageal cancer cell lines **A.** The chemical molecular formula of YM155. **B.** Whole cell protein extract from each of eight esophageal cancer cell lines was subjected to analyzed by western blotting using antibodies against survivin and SLC35F2. Quantitative values of relative survivin and SLC35F2 levels normalized to actin (mean ± SD). **C.** Dose-response curve for KYSE410, KYSE150, and MEF cells after YM155 treatment for 24 h. Cells were treated with the indicated concentrations of YM155 for 24 h. The survival curves of KYSE 410, KYSE150 and MEF cells were constructed using the CCK-8 assay. **D.** necrosis was measured as LDH release after YM155 treatment for 24 and 48 h in both KYSE410 and KYSE150 cells. **E.** The long-term viability of cells was determined after YM155 treatment for 24 h in KYSE410 and KYSE150 cells using the colony-formation assay.

Previous work revealed that the expression level of the transporter SLC35F2 is associated with sensitivity to the drug YM155 [22]. To assess the expression of SLC35F2 and survivin, a panel of eight ESCC cell lines was constructed for evaluation via western blot analysis. The molecular formula of Sepantronium bromide (YM155) is shown in Fig. 1A. It shows that the expression levels of SLC35F2 and survivin vary across cell lines in Fig. 1B, with KYSE140, KYSE150, KYSE170, KYSE410 and WHCO1 showing relatively high expression of SLC35F2 and survivin and KYSKE30, KYSE180 and KYSE510 showing relatively low expression levels of SLC35F2. Based on the cell panel data, the cell lines KYSE410 and KYSE150 were chosen for further investigation of the efficacy of YM155. Both KYSE150, which has high survivin expression, and KYESE410, which has more modest expression, have high SLC35F2 expression. The MEF cell line was used as a control. To assess the efficacy of YM155, both cell lines were treated with different concentrations of YM155 for 24 or 48 h. Dose-response experiments (Fig. 1C) and Time-dependent cure (Fig. S1) demonstrate that both cell lines were exquisitely sensitive to YM155, whereas MEF cells showed no response to YM155 at concentrations below 100 nM. We also found that YM155 markedly suppressed colony formation compared with untreated cells (Fig. 1E).

**Figure 2:**
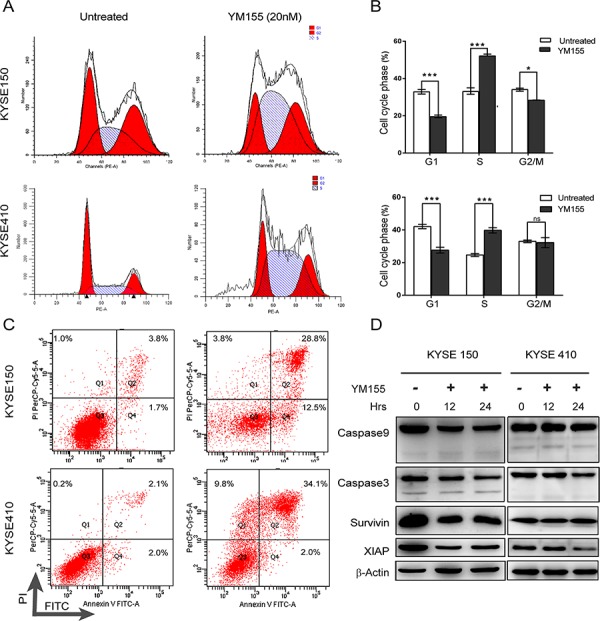
YM155 triggers non-apoptotic cell death in KYSE410 and KYSE150 **A.** The cell-cycle analysis of KYSE410 and KYSE150 cells was performed using PI staining and analyzed by flow cytometry after 12 h of YM155 treatment. **B.** A histogram illustrated the percentages of KYSE410 and KYSE150 cells in G1, S and G2/M phases. **C.** Apoptosis, as quantified by annexin V/PI staining. KYSE410 and KYSE150 cells were treated with YM155 (20 nM) for 24 h. Cells were stained by annexin V/PI and analyzed by flow cytometry. **D.** KYSE410 and KYSE150 cells were treated with 20 nM YM155 for 12 or 24 h. Total cell lysates were assayed for caspase-9 and caspase-3 cleavage and for survivin and XIAP protein expression by western blotting. Beta-actin was used as a loading control.

To define the response to YM155, the cell-cycle profiles of cells treated with YM155 for 12 h were determined by flow cytometry (Fig. 2A and 2B). As shown in Fig. 2A and 2B, 12 h of exposure to YM155 changed the cell-cycle phase distribution of KYSE410 and KYSE150. Significantly increased proportions of cells in S phase were detected in both KYSE410 and KYSE150 after YM155 treatment for 12 hours. However, the proportion of cells in G1 phase markedly decreased after YM155 treatment. Taken together, these data suggest that both KYSE410 and KYSE150 esophageal cancer cell lines are sensitive in response to YM155 treatment.

### YM155 causes non-apoptotic cell death in esophageal cancer cells

**Figure 3:**
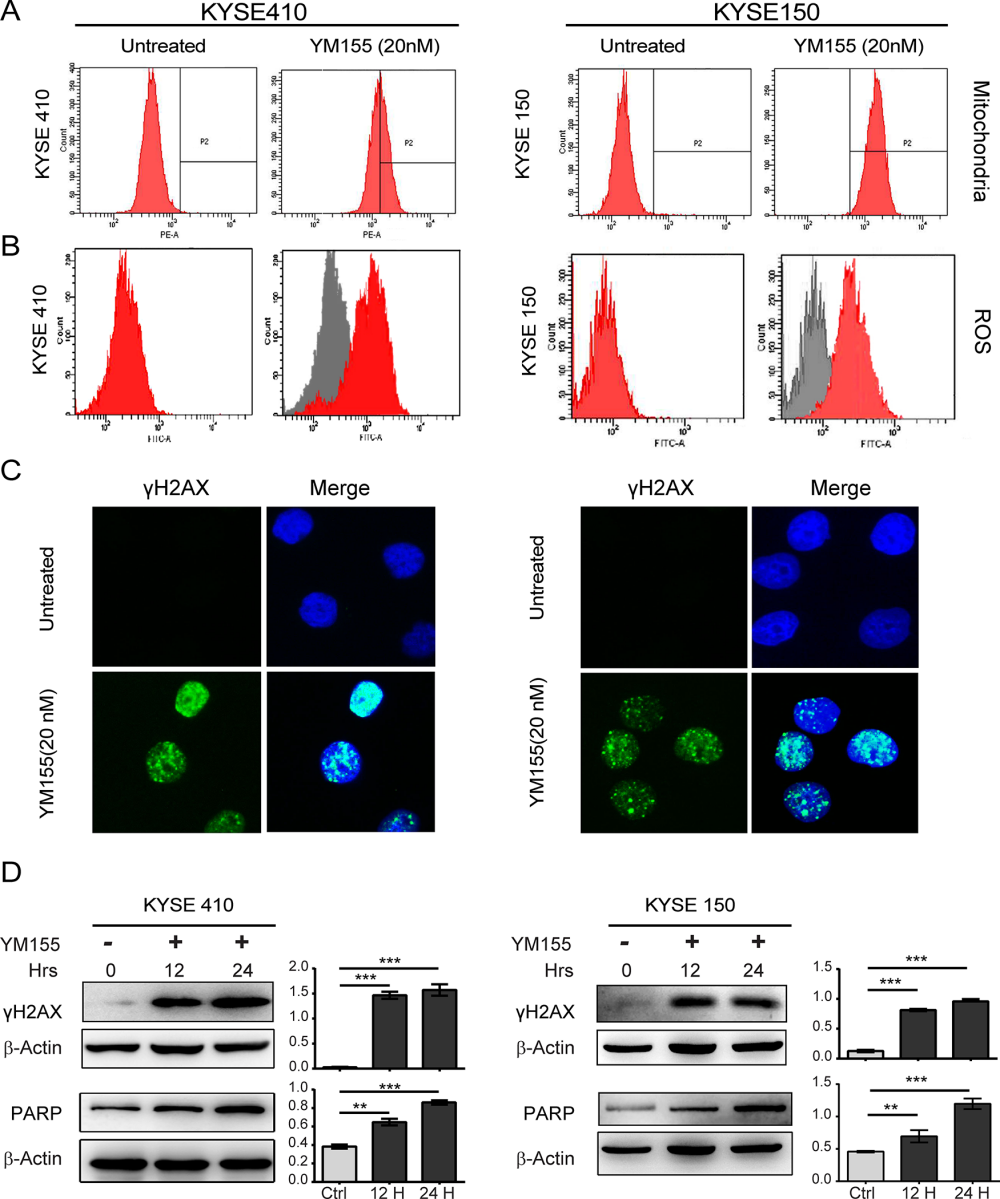
YM155 induces DNA damage and ROS production in both KYSE410 and KYSE150 cells **A.** KYSE410 and KYSE150 cells were treated with 20 nM YM155 for 12 h, stained with Mito Tracker Red CMXRos, and analyzed by flow cytometry. **B.** KYSE410 and KYSE150 cells were treated as indicated for 12 h. Cells were stained with H2DCF-DA for 20 min, and ROS were measured by flow cytometry. All experiments were conducted at least three times. **C.** Localization of gamma-H2AX in KYSE410 and KYSE150 cells was determined with immunofluorescent analysis after 12 h YM155 treatment. Nuclei were stained with DAPI, as shown in blue. Scale bars: 10 μm. **D.** Total cellular protein extract from KYSE410 and KYSE150 after YM155 treatment for 12 and 24 h was subject to analysis by western blotting using antibodies against gamma-H2AX. Quantitative values of relative gamma-H2AX levels on the right are normalized to actin (mean ± SD).

To test whether the reduced cell viability of esophageal cancer cell lines following YM155 treatment is associated with apoptosis, both KYSE410 and KYSE150 cells were analyzed by flow cytometry using annexin V-FITC and propidium iodide (PI). As shown in Fig. 2C, YM155 treatment for 24 h increased the fraction of cells with PI and annexin V double-positive staining in KYSE410 and KYSE150 cell lines. To further investigate whether YM155 treatment induces cell death via apoptosis, mitochondrial membrane potential, ROS and the active formation of caspases was examined by flow cytometry and western blot analysis. The mitochondrial membrane potential was not decreased after 12 h of YM155 treatment (Fig. 3A), and only a small increase in ROS was detected after 12 h of YM155 treatment in KYSE410 and KYSE510 cells (Fig. 3B). Treatment with YM155 for 12 or 24 h did not affect the cleavage of caspase-9 and caspase-3. The levels of survivin and XIAP, an anti-apoptotic marker, were also measured after YM155 treatment. Survivin expression decreased after YM155 treatment in KYSE150 cells but not KYSE410 cells, whereas XIAP protein levels greatly decreased in both cell lines (Fig. 2D). Upon further analysis using transmission electron microscopy (TEM), KYSE410 cells treated with YM155 for 24 h displayed swollen mitochondria and discontinuous cytoplasmic membranes but lacked typical apoptotic features such as condensed nuclei, plasma membrane blebbing and apoptotic bodies (Fig. 5E). Collectively, these results indicate that YM155 predominantly induced a non-apoptotic, caspase-independent form of cell death in KYSE410 and KYSE150 cells.

**Figure 4:**
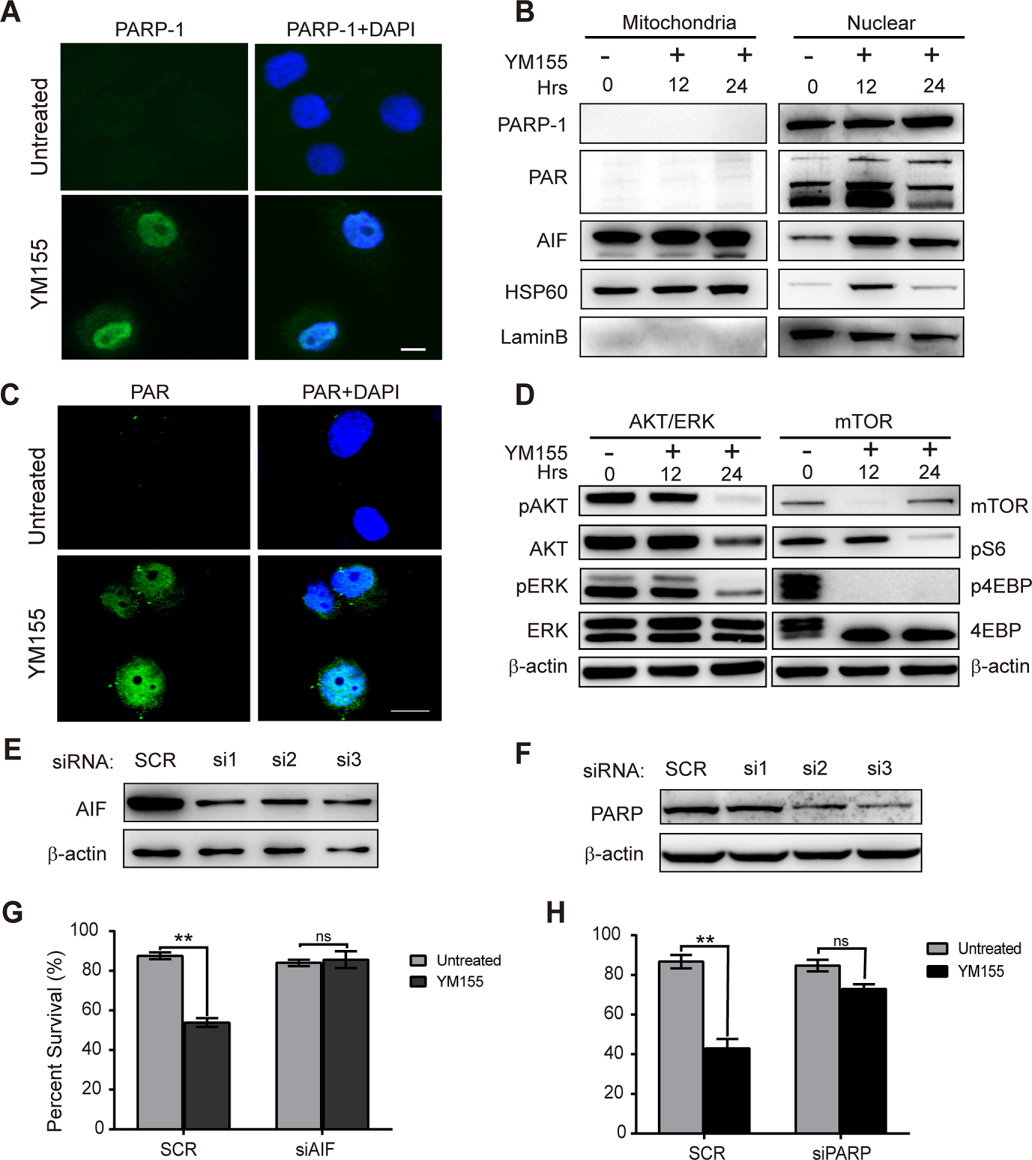
YM155 induces PARP-1-dependent parthanatos **A.** Nuclear accumulation of active PARP-1 in KYSE410 cells after 12 h of YM155 treatment was evaluated using immunofluorescent analysis. Nuclei were stained with DAPI, as shown in blue. Scale bars: 10 μm. **B.** Following treatment with YM155 for 12 h, cytosolic fractions were isolated from the treated cells and analyzed for PARP-1, PAR and AIF by western blotting. HSP60 and Lamin B protein were used as loading and fraction controls, respectively. **C.** Accumulation of the poly-ADP polymer in KYSE410 was evaluated using immunofluorescent analysis after YM155 treatment for 12 h. Nuclei were stained with DAPI, as shown in blue. Scale bars: 10 μm. **D.** Total KYSE410 cell protein extract was analyzed for phosphorylation of AKT and ERK, mTOR and phosphorylation of S6 and 4-EBP by western blotting. Beta-actin was used as a loading control. **E** and **F.** The effects of *PARP* and *AIF* gene siRNA knockdown were analyzed by western blot analysis. **G** and **H.** After treatment with YM155, the survival curve following *PARP* and *AIF* knockdown in KYSE410 cells was detected using the CCK-8 assay.

Although YM155 has been reported to induce caspase activation [23], the active cleavage of caspases was not detected in this study. It was not known whether YM155 treatment induces other factors that trigger cell death. Recent data have shown that YM155 induces autophagy-dependent cell death in salivary adenoid cystic carcinoma [24]. Autophagy markers, including LC3I, LC3II, Beclin and BNIP3, were measured by western blot analysis. As shown in Fig. S2, YM155 treatment did not produce time-dependent increases in LC3I, LC3II, Beclin and BNIP3 levels in KYSE410 cells, suggesting that YM155 did not induce autophagy. To explore whether the mTOR pathway changed, we examined mTOR pathway-associated proteins including mTOR, AKT, S6, and ERK. The expression levels of mTOR, and the phosphorylation of AKT, S6, 4-EBP and ERK decreased after treatment with YM155 in KYSE410 but not KYSE150 cells, indicating that the decrease in the mTOR pathway may be associated with YM155-induced cell death (Fig. 4D).

### YM55 induces DNA damage and PARP activity

Previous results have demonstrated that YM155 generates DNA damage and mediates DNA damage toxicity in a human myeloid leukemia cell line *in vitro* and *in vivo* [22]. We therefore assessed canonical DNA damage after treatment with YM155 for 12 or 24 h in KYSE410 and KYSE150 cells using immunofluorescence and western blot analysis for γH2AX. As shown in Fig. 3C and 3D, we observed greatly increased nuclear expression of γH2AX after 12 h of YM155 treatment in both KYSE410 and KYSE150 cell lines. A significant induction of γH2AX expression was detected in both cell lines by western blot analysis at the 12- and 24-h time points, indicating the presence of DNA double-strand breaks.

Poly (ADP-ribose) polymerase 1 (PARP-1) is an important nuclear enzyme that responds to DNA damage and not only plays a pivotal role in DNA repair but also, as a marker of DNA damage, contributes to other aspects of nucleic acid metabolism, including transcriptional regulation [25, 26]. To further assess DNA damage after YM155 treatment, KYSE410 and KYSE 150 cells exposed to 20 nM YM155 for 12 or 24 h were evaluated by western blot analysis using an antibody recognizing both full-length and cleaved PARP. As shown in Fig. 3D, treatment YM155 for either 12 or 24 h led to the significant time-dependent accumulation of full-length PARP. Quantification of the ratio between PARP and actin in KYSE410 and KYSE150 cells is provided in Fig. 3D. Taken together, these data indicate that the loss of cellular viability in esophageal cancer cell lines after YM155 treatment is associated with YM155-mediated DNA damage.

### PARP and AIF are required for YM155-induced parthanatos cell death

It has been reported that massive DNA damage and PARP1 activation can induce a specific form of cell death termed “parthanatos,” which is morphologically characterized by poly-ADP formation, the release of apoptosis-inducing factor (AIF) from the mitochondria, nuclear translocation of AIF and membrane rupture [27–29]. We have previously shown that full-length PARP-1 protein levels in KYSE410 cells greatly increased after YM155 treatment for 12 and 24 h. To determine whether PARP1 was extensively activated after YM155 treatment in KYSE410 cells, we analyzed the expression of PARP1 and PAR by immunofluorescence and western blot analysis after YM155 treatment. As shown in Fig. 4A, compared with untreated cells, YM155 treatment induced the accumulation of PARP1 in the nuclei. To further analyze PARP1 activation, total cytosolic fractions were isolated and tested for expression of PARP1 and PAR by western blot analysis. Significant accumulation of PARP-1 and PAR was observed in the nuclear fraction of KYSE410 cells after treatment with YM155 for 12 or 24 h (Fig. 4B). Immunofluorescence and western blot assays using a specific anti-PAR antibody confirmed that Poly-ADP is formed in the cytoplasm and nuclei of KYSE410 cells, indicating that PARP is activated in the nuclei after treatment (Fig. 4C).

PARP activation results in the release of AIF from mitochondria and occurs directly through PAR targeting the mitochondrial membrane, and translocation of AIF into the nucleus plays an essential role in parthanatos cell death [30, 31]. Therefore, to further explore the PARP1-AIF pathway, we also assessed AIF release and translocation after treatment with YM155 for 12 and 24 h using immunofluorescence and western blot analysis. Following treatment with YM155, immunoblotting and immunofluorescence results indicated that endogenous AIF translocated into the nucleus in a time-dependent manner, leading to parthanatos cell death in KYSE410 cells (Fig. 4B and Fig. S3). To investigate the roles of PARP1 and AIF in YM155-induced parthanatos cell death, siRNA targeting PARP-1 and AIF were used to knock down PARP-1 and AIF expression in KYSE410 cells. After siRNA transfection, knockdown of PARP-1 and AIF was analyzed by western blotting. Compared to the control group, PARP-1 and AIF protein expression were found to decrease at 24 h after siRNA transfection in KYSE410 cells (Fig. 4E and 4F). The scramble control group and targeted siRNAs group of KYSE410 cells were treated with YM155 for 24 h, and cell survival was analyzed with the CCK-8 viability assay. As shown in Fig. 4G and 4F, the effect of YM155 was inhibited by PARP-1 or AIF knockdown using siRNA transfection in KYSE410 cells. Collectively, these data indicate that PARP-1 and AIF play important roles in PARP-1-mediated parthanatos cell death after YM155 treatment in KYSE410 cells.

### Gene expression changes in YM155-treated KYSE410 cells

**Figure 5:**
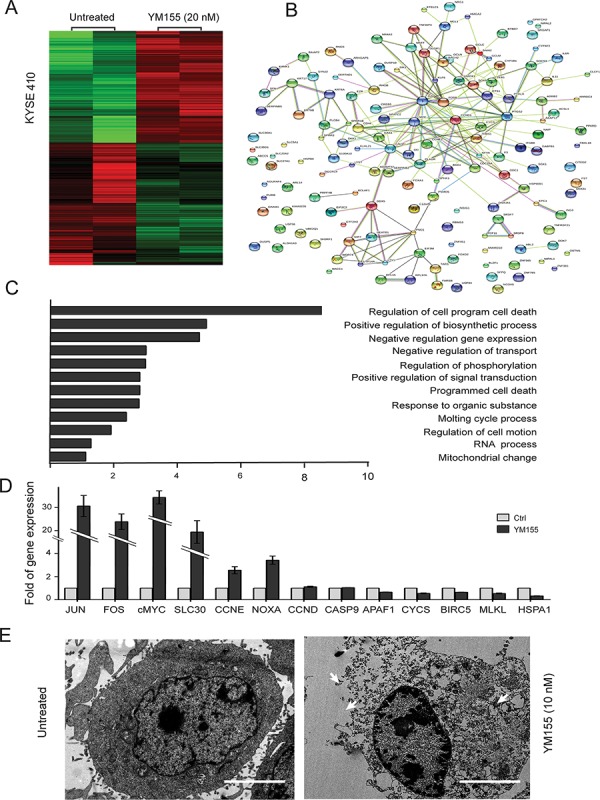
Clustering display of the microarray data after YM155 treatment **A.** RNA was extracted using an RNA isolation kit for microarray analysis after YM155 treatment for 6 h in KYSE410 cells. Heatmap representing genes upregulated and downregulated in the micro-array analysis of KYSE410, demonstrating gene categories with altered expression after YM155 treatment. Data are represented as log2 fold change, *n* = 2 per group. **B.** The upregulated genes were functionally analyzed using the online tool STRING. **C.** The upregulated genes were functionally classified based on their biological process using the DAVID functional annotation clustering tool. **D.** The mRNA levels of cell death-associated and candidate genes in KYSE410 treated with 20 nM YM155 for 6 h was determined using real-time RT-PCR. Data represent the mean ± SEM of relative mRNA levels versus untreated cells. **E.** Transmission electron microscopy of KYSE410 cells after YM155 treatment. The integrity of the membrane was noted in the untreated cells, and the collapse of the membrane and the swelling of the cellular organelles were observed in cells treated with YM155.

To further investigate the mechanism of YM155-induced PARP-mediated parthanatos cell death in KYSE410 cells, Affymetrix Human Genome U133 plus 2.0 arrays were used to analyze mRNA changes in YM155-treated and untreated KYSE410 cells. Each experiment included two biological replicates. The array data were analyzed using Significance Analysis of Microarrays (SAM 3.02) software. The cutoff criteria for significantly differentially expressed genes were set to a ratio of >2-fold difference in expression and an adjusted *P* value of < 0.05. In the YM155-treated group, a total of 549 genes fulfilled these stringent cutoff criteria; 221 genes were upregulated, whereas 328 genes were downregulated. Fig. 5A shows a heatmap represented of different genes expression generated from Affymetrix chips of untreated and YM155-treated cells. Within the upregulated genes, similar annotation terms are grouped into clusters using DAVID functional annotation clustering to measure the relationships between the annotation terms and their co-association with the genes [20]. It is important to note that the upregulated genes were predominantly involved in the regulation of programmed cell death, the positive regulation of biosynthetic processes and the negative regulation of gene expression (Fig. 5C). Using the online database resource Search Tool for the Retrieval of Interacting Genes (STRING), we evaluated the interactions between the upregulated genes following YM155 treatment in KYSE410 cells [21]. As shown in Fig. 5B, we identified certain vital core genes from STRING, including FOS, JUN, c-MYC, and CCNE, which are regulated by activated PARP [32, 33]. Canonical biological process of genes up-regulated in KYSE410 is available in Supplementary Tables 2. To better understand how KYSE410 cells execute apoptosis or other cell death after YM155 treatment, real-time PCR was conducted on genes associated with programmed cell death, such as FOS, JUN, c-MYC, CCNE, CCND, NOXA, MLKL, BIRC5, CYCS, APAF1, CASP9, SLC30A2 and HSPA1A. The mRNA levels of FOS, JUN, c-MYC, SLC30A2, CCNE, and NOXA were significantly elevated in KYSE410 cells after treatment with YM155 for 6 h. Furthermore, the mRNA levels of MLKL, BIRC5, CYCS, APAF1, CASP9 and HSPA1A, which are associated with apoptosis and necrosis, remained unchanged or slightly decreased in the YM155-treated KYSE410 cells (Fig. 5D). Therefore, transcript analysis demonstrates that YM155 treatment in KYSE410 cells facilitates the transcription of active PARP-regulated genes such as FOS, JUN, and c-MYC but not of apoptotic or necrotic genes.

### *In vivo* efficacy of YM155 on esophageal cancer cell lines

**Figure 6:**
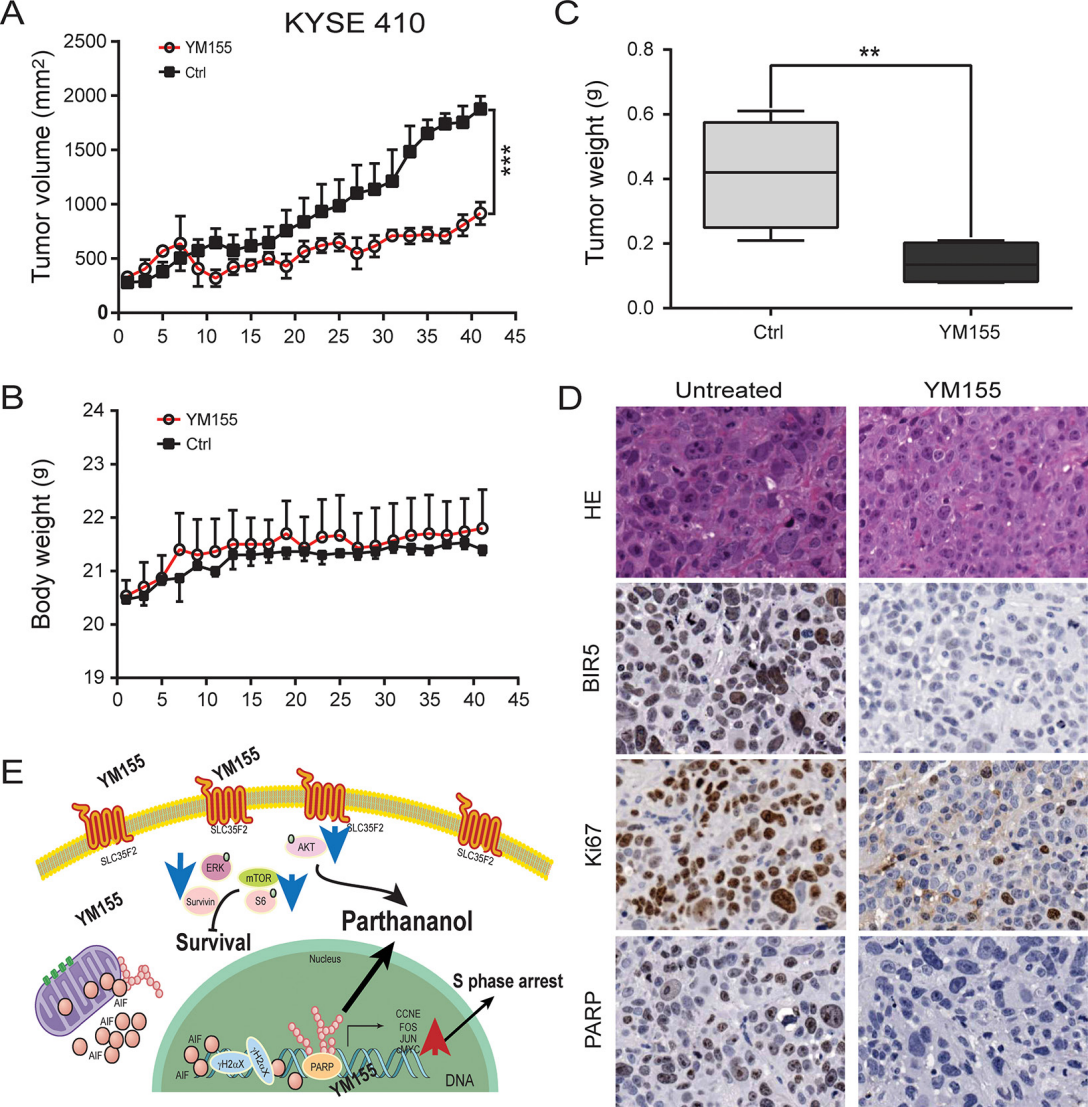
Inhibition of tumor growth *in vivo* **A.** Tumor volume was measured every 2 to 3 days after YM155 treatment. KYSE410 cells decreased the growth of the xenograft tumors compared to the untreated group (*P* < 0.001). **B.** Mouse body weight was measured over the course of 45 days. YM155 did not affect mouse body weight. **C.** Tumor weight was significantly reduced at study termination (*P* < 0.05). **D.** Xenograft tissue was subject to analysis by immunohistochemistry for survivin, Ki67 and PARP (× 200). Survivin, Ki67 and PARP localized mainly in the nuclei of esophageal cancer epithelial cells. **E.** Illustration of the signaling pathway for YM155-induced PARP-mediated parthanatos. Innate hyperactive PARP and AIF translocation are required for PARP-1-dependent parthanatos induced by YM155. The blue and red arrows represent the downregulated and upregulated genes, respectively.

Having shown that YM155 treatment induces PARP-1-dependent parthanatos cell death in esophageal cancer cells *in vitro*, we next investigated the efficacy of YM155 in inhibiting KYSE410 and KYSE150 tumor growth in a mouse xenograft model of esophageal cancer. Mice bearing KYSE410 or KYSE150 tumors were inoculated for 10 days by intraperitoneal injection (i.p.) with 5 mg/kg YM155 respectively, in the YM155 treatment group (*n* = 5 for KYSE410 and KYSE150) or with 0.9% normal saline for the controls (*n* = 5 for KYSE410 and KYSE150). As shown in Fig. 6A, the average tumor burden at the beginning of the treatment was not significantly different between the YM155-treated and control groups. YM155 treatment resulted in significant antitumor activity and reduced the growth of KYSE410 or KYSE150 xenograft tumors compared with the untreated group (Fig. S4A). We observed that there was no obvious toxicity in the various groups in that the mean body weight was similar in all groups (Fig. 6B and Fig. S4B). There was a 66.3% reduction in tumor weight vs. the control group (*P* < 0.05) in the KYSE410 xenograft model and a 78.3% reduction in the KYSE150 xenograft model respectively (Fig. 6C and Fig. S4C).

To assess the biological effects of YM155-based therapy, tumors from KYSE410 and KYSE150 models were examined for markers of PARP-1, tumor cell proliferation (Ki67), and survivin. Changes in these markers reflected antitumor activity in response to YM155. More specifically, significant reductions in survivin, Ki67 and PARP were observed in the KYSE410 model after treatment with KYSE155 (Fig. 6D).

As illustrated in Fig. 6E, we propose a model to explain how YM155 induces PARP-mediated parthanatos cell death in ESCC cells: (i) the hyper-activation of PARP-1, leading to PAR and AIF translocation, (ii) the requirement of PARP-1 and AIF for YM155-induced necrosis, and (iii) the inhibition of mTOR pathways. These results show that PARP-1-mediated parthanatos cell death plays a critical role and is essential for the efficacy of YM155 treatment in esophageal cancer therapy.

## DISCUSSION

We examined the therapeutic potential of YM155 in esophageal cancer both *in vitro* and *in vivo*. Previous studies have implicated apoptosis in YM155-induced cytotoxicity in multiple types of cancer [23, 34, 35]. The initial screen identifying YM155, using an artificial reporter system including the survivin promoter, was conducted in human cervical epithelia carcinoma cells (HeLa) and Chinese hamster ovary cells [5]. YM155 efficiently repressed survivin promoter activity via binding and disrupting proteins such as interleukin enhancer-binding factor-3 (ILF3-p54/nrb complex) and SP1 [5, 9, 36, 37]. In this study, we found that YM155 partly reduced survivin expression in KYSE150 but not KYSE410 cells. YM155 induced DNA damage response including γH2AX and PARP-1 hyper-activation. Our results are consistent with Gloro and Kasap's reports that YM155 induced DNA damage and cell death [10, 38]. For the first time, YM155-induced PARP-1-dependent parthanatos cell death has been observed in esophageal cancer cells, and the underlying cytotoxic mechanism is tightly linked to PARP-1 hyper-activation leading to PAR formation and AIF translocation. Thus, YM155 is a novel inducer of PARP-1-dependent parthanatos cell death and has potential as an effective esophageal cancer treatment.

Cell death can be enacted through various mechanisms including apoptosis, necrosis, and autophagy. In previous studies, we demonstrated that apoptotic, necrotic and autophagic cell death occurs after anticancer drug treatment in esophageal cancer cells [16–18]. Although these three distinct modes of cell death were considered to share common biochemical features for death execution, recent advances have revealed parthanatos cell death, a new variant occurring in many neurodegenerative disorders, which is reported to be PARP-1-dependent and caspase-independent [29, 31, 39, 40]. PARP-1 takes part in genomic repair under physiological condition and is considered a “genome guardian” [41, 42]. Mild DNA damage induces PARP-1 activation to repair the damaged DNA, whereas excessive activation of PARP-1 leads to a unique form of cell death termed parthanatos that is largely mediated via accumulation of poly-ADP polymers and the translocation of AIF from the mitochondria [40, 43–45]. In this study, we found that YM155 selectively induces parthanatos cell death in KYSE410 and KSYE150 cells. This conclusion is based on the following observations in KYSE410 and KYSE150 cells undergoing YM155-induced cell death: (i) little nuclear fragmentation or caspase cleavage; (ii) the release of LDH, PARP-1 hyper-activation, poly-ADP polymer formation and translocation of AIF and (iii) the visualization of disrupted cytoplasmic membranes and cellular organ swelling using TEM. Furthermore, our data show that YM155 led to a significant time-dependent accumulation of full-length PARP after YM155 treatment. Poly-ADP polymer formation was measured in the cytosol and the nucleus of KYSE410 cells after YM155 treatment, using a specific anti-PAR antibody for immunofluorescence and western blot analysis. In addition, knockdown of PARP1 using siRNA interference greatly reduced YM155-induced PARP-1 mediated parthanatos cell death in KYSE410 cells. Furthermore, we demonstrate that AIF suppression by siRNA decreases the antitumor activity of YM155 in KYSE410 cells. Collectively, these results suggest that YM155-mediated PARP-1 hyper-activation enhances the antitumor effect through the formation of poly-ADP polymers as well as the translocation of AIF, ultimately leading to parthanatos cell death. Previous results suggested that parthanatos cell death is a result of intracellular energy depletion, resulting from excess nicotine amide adenine dinucleotide (NAD+) and ATP consumption [28, 46]. Consumption of NAD+ as a PARP-1 substrate generates poly-ADP polymer, which translocates to the cytosol to induce cell death via the nuclear translocation of AIF to effect large-scale DNA fragmentation [47, 48].

Although apoptosis is the predominant pathway of YM155-induced cell death in cancer cells, it is reported that autophagy-dependent cell death presents an alternative YM155-effected pathway in salivary adenoid cystic carcinoma [24]. The current study shows that both apoptotic and necrotic machinery were attenuated during YM155-induced parthanatos cell death in KYSE410 and KYSE150 cells. Microarray analysis demonstrated that the transcription of BIRC5, CYCS, APAF1, CASP9, HSPA1A and MLKL, which are associated with apoptosis and necrosis, were decreased in YM155-treated KYSE410 cells. Furthermore, autophagy-associated markers, including LC3I, LC3II, Beclin and BNIP3 [18, 49], did not increase in a time-dependent manner after YM155 treatment in KYSE410 cells, suggesting that YM155 did not induce autophagy. On the contrary, our results indicate that the mRNA levels of FOS, JUN, c-MYC and SLC30A2 in KYSE410 cells were elevated more than 20-fold after treatment with YM155 for 6 h. Mostocotto's study showed that PARP-1 plays an essential role in regulating the expression of c-MYC, c-FOS, and JUN. Active PARP-1 enhanced several changes involved in binding to functionally relevant sequences of the promoter, chromatin relaxation, the exchange of transcription factors and the accumulation of phosphoacetylated histone H3, consequently facilitating the induction of c-MYC [32]. Several studies have shown that PARP-1 acts as a regulator of various transcriptional factors, such as E2F-1, FOXO1 and FOS, and of genomic methylation patterns [33, 50, 51]. In the present study, we observed significantly increased transcription of c-MYC, c-FOS, and JUN in microarray analysis and PCR validation experiments. Based on transcription of upregulated genes, we further confirmed the excessive activation of PARP-1 upon YM155 treatment in esophageal cancer cells.

This is the first study demonstrating that YM155 causes hyper-activation of PARP1 and induces PARP-1-dependent parthanatos cell death. Previous neuronal death studies have shown that parthanatos cell death is a programmed necrotic demise, characteristic of ATP consumption due to NAD+ depletion by PARP-1-mediated PAR formation on target proteins [31, 43, 45]. Further study is necessary to determine (i) why in esophageal cancer, YM155 initiates PARP-1 hyperactivation-mediated Parthanatos cell death as opposed to caspase-dependent apoptosis; (ii) how poly-ADP in the cytosol induces a death-signaling cascade and executes biological functions in the PARP-1-mediated pathway and; (iii) the identity of proteins targeted by PAR to explore the complex pathway from nuclear to mitochondria during the process of parthanatos.

In conclusion, we report that YM155 treatment can kill esophageal cancer cells by causing DNA damage, PARP-1 hyper-activity, and AIF translocation leading to PARP-1-dependent parthanatos cell death and that it reduced tumor burden in both *in vitro* and in animal models *in vivo* of esophageal cancer. Our preclinical results provide a rationale for future clinical investigation of the therapeutic efficacy of YM155 in patients with esophageal cancer.

## MATERIALS AND METHODS

### Cell culture and reagents

The esophageal cancer cell lines KYSE410 and KYSE150 were obtained from Dr. Yutaka Shimada at the Hyogo College of Medicine [15]. Mouse embryonic fibroblasts (MEFs) were obtained from Dr. Zhang, Hongbin at Peking Union Medical College. All cell lines were grown in RPMI supplemented with 10% FBS, 100 U/ml penicillin, and 100 μg/ml streptomycin at 37°C in 5% CO_2_.

Sepantronium Bromide (YM155) was purchased from Selleck Chemicals (Houston, TX, USA), dissolved in dimethyl sulfoxide (DMSO) to 10 mmol/L stock solutions, and diluted with culture medium. MitoTracker Red CMXRos was purchased from Invitrogen (Life Technologies, USA) and 2, 7-dichlorofluorescein diacetate (H2DCF-DA) and 40, 6-diamidino-2-phenylindole (DAPI) from Sigma-Aldrich (St. Louis, Missouri, USA). Anti-caspase-3 and anti-caspase-9 antibodies were purchased from Enzo (Life Sciences, NY); γH2AX, Survivin, XIAP, mTOR, AKT, Phospho-AKT, ERK, Phospho-ERK, Phospho-S6, 4EBP1, Phospho-4EBP1, LC3, Beclin and Survivin antibodies were purchased from Cell Signaling Technology Inc. (Danvers, MA, USA); Apoptosis-inducing factor (AIF) antibody from Santa Cruz Biotechnology, (Santa Cruz, CA, USA); SLC35F2 antibody from Abcam (Cambridge, MA, USA); PAR and PARP antibodies from BD Pharmingen (BD Biosciences, San Jose, CA); and β-actin antibody from Sigma-Aldrich (St. Louis, Missouri, USA).

### *In vitro* assays for cell viability, colony formation, cell-cycle progression and cell death

Cells were grown in 96-well plates to 20–30% confluence before treatment with YM155. After YM155 treatment for 24 or 48 h, cell viability was assessed with the CCK-8 assay (Dojindo, Japan), and the absorbency value was read using a Model 680 microplate reader (Bio-Rad, CA) according to the manufacturer's instructions. For the colony-formation assay, cells were seeded in 6-well plates at a density of 5 × 10^5^ cells per well and treated with YM155 for 24 h. Cells were then washed 3 times with PBS, and media was replaced with fresh RPMI containing 10% FBS. After 7 days, cells were stained with 0.25% crystal violet (AMRESCO) for 20 min. All experiments were conducted at least 3 times.

Cell-cycle distribution was determined using a cell-cycle detection reagent kit (4A Biotech, Beijing, China). Cells were fixed with ice-cold 70% ethanol and incubated at 4°C. Ethanol-fixed cells were stained with propidium iodide (PI) and analyzed by flow cytometry. Cell death was detected by annexin V and propidium iodide (PI) staining using apoptosis detection kits (Biosea Biotechnology, Beijing, China), coupled with flow cytometry as described previously [16]. For data collection, 10, 000 cells were acquired per sample and analyzed using BD software. Mean values were obtained from 3 independent assays.

### Mitochondrial membrane permeability and ROS assay

KYSE410 and KYSE150 cells grown in RPMI medium supplemented with 10% FBS were treated with YM155 for 12 h, washed with PBS and incubated with the mitochondria-specific fluorescent probe MitoTracker Red for mitochondrial membrane permeability (MMP) detection and with 2′, 7′-dichlorofluorescein diacetate (H2DCF-DA) for the ROS assay. ROS and MMP activation were analyzed using flow cytometry according to the manufacturer's protocol (EPICS ELITE ESP, USA) to calculate the percentage of positive cells.

### Measurement of LDH in culturing medium by ELISA

Cells grown in 10-cm dishes were treated with YM155 for 24 or 48 h, as indicated in the figure legends. The culture medium was collected and analyzed for LDH using a lactate dehydrogenase (LDH) ELISA kit (Abcam) according to the manufacturer's protocol.

### Western blot analysis

Following YM155 treatment, cells were cultured in 10-cm plates for 12 or 24 h. Cells were harvested and the nuclear, cytoplasmic and mitochondrial protein fractions were separated by ProteoExtract Subcellular Proteome Extraction Kit (Calbiochem, Darmstadt, Germany) according to the manufacturer's instructions. Total protein lysate or cellular fraction protein (10–20 μg) from each sample was separated by SDS-PAGE, transferred to polyvinylidene difluoride (PVDF) membranes, and used for western blot analysis, as described previously [17]. Antibodies against the following proteins were used: caspase-9, caspase-3, survivin, γH2AX, XIAP, mTOR, AKT, Phospho-AKT, ERK, Phospho-ERK, Phospho-S6, 4EBP1, Phospho-4EBP1, LC3, Beclin, Apoptosis-inducing factor (AIF), SLC35F2, PAR, PARP and β-actin.

### Immunofluorescent staining

Cells grown on glass coverslips were fixed in 4% paraformaldehyde for 30 min after YM155 treatment, rinsed 3 times with PBS, permeabilized with 0.1% Triton X-100, and blocked for 30 min with PBS containing 2% BSA. The cells on coverslips were incubated for 1 h at room temperature with the indicated primary antibodies and then incubated at room temperature for 30 min with the appropriate fluorescent secondary antibodies. Fluorescence images were captured with a Nikon ECLIPSE 80i microscope [18].

### Small interfering RNA (siRNA) transfection

For PARP-1 and AIF knockdown, KYSE410 cells grown in a 6-well plate were transfected with PARP-1 and AIF siRNA using Lipofectamine (Invitrogen, USA) according to manufacturer's instruction [17]. The siRNAs targeting AIF were siRNA-1: 5′-CCACCUUCUUUCUAUGUCUTT-3′, siRNA-2: 5′-GGGCACAGAAGUGAUUCAATT-3′, and siRNA-3: 5′-CUGCAUGCUUCUACGAUAUTT-3′, and the siRNAs targeting PARP-1 were siRNA-1: 5′-GAGACCC AAUAGGCUUAAUTT-3′, siRNA-2: 5′-GAGCACUUCAUGAAAUUAUTT-3′, and siRNA-3: 5′-GAGGAAGGUAUCAACAAAUTT-3.′ Nonspecific small interfering RNA (scramble, SCR) oligonucleotides were synthesized at GeneChem Inc., dissolved in PBS, and diluted with culture medium.

### Microarray and real-time PCR analysis

Total RNA was isolated from untreated and YM155-treated KYSE410 cells using an RNeasy^®^ mini kit (Qiagen). The quality of the total RNA was determined using a NanoDrop spectrophotometer (ND-2000; Thermo Fisher Scientific). RNA with an A260/A280 absorbance ratio ranging from 1.8 to 2.0 was used for cDNA synthesis. Gene expression profiles were analyzed on a GeneChip^®^ Human Genome U133 Plus 2.0 array (Affymetrix, Santa Clara, CA), which contains 54, 000 probe sets representing approximately 47, 000 genes. The signal intensity of the gene expression was analyzed to generate CEL files using the default setting of Affymetrix^®^ GeneChip^®^ Command Console^®^ 3.2 (AGCC) Software. The Affymetrix Microarray Suite 5.0 (MAS5) and the Robust Multi-array Average (RMA) algorithm were used for the expression summary and signal calculation of the GeneChip^®^ Human Genome U133 2.0 data [19], respectively. Differentially expressed genes were selected based on *a* > 2.0-fold change and a *q* value < 5%. The Entrez gene identifiers were used to perform enrichment analysis using the Database for Annotation, Visualization and Integrated Discovery (DAVID) and the online database resource Search Tool for the Retrieval of Interacting Genes (STRING) [20, 21].

Total RNA was isolated from the untreated and YM155-treated KYSE410 cells, and first-strand reverse transcription was performed using the SuperScript^®^ III Reverse Transcriptase kit (Invitrogen, USA). Primers were designed with Primer-Blast software (http://www.ncbi.nlm.nih.gov/tools/primer-blast/). Amplification reactions were conducted using the SsoFast™ EvaGreen^®^ Supermix with a CFX 96™ real time system (Chemoscience, USA). The complete list of gene-specific real-time primers used in this study is available in supplementary Table 1. GAPDH served as an internal control to normalize the loading of the template cDNA. Each experiment was repeated at least twice, and the fold change in gene expression was assessed using the ΔCt method[16].

### Transmission electron microscopy

Cells grown in 10-cm dishes were treated with YM155 for 24 h, fixed for 2 h with 2.5% glutaraldehyde in 0.1 M sodium cacodylate buffer (pH 7.4), further fixed in osmium tetroxide solution for 1 h, and subjected to electron microscopy analysis with a JEM-2100 electron microscope (JEOL, Japan).

### *In vivo* xenograft assay

Animal experiments were carried out as previously described [17]. Briefly, 5 × 10^6^ KYSE410 or KYSE150 cells were suspended in 100 μl PBS and injected subcutaneously into the right flanks of female nude mice (*n* = 10). When a tumor was detectable (approximately 14 days after subcutaneous injection), mice were randomly assigned to two groups, receiving either YM155 or 0.9% saline. The YM155 treatment group was given continual intraperitoneal injections for 10 days. YM155 was dissolved in PBS to a working concentration of 5 mg/kg. To monitor tumor growth, the tumor size was measured every 2–3 days using digital calipers. Tumor volume was calculated using the formula: 0.5 × *a* × *b*^2^, where *a* is the length of the tumor and *b* is the width. All experimental procedures using animals were previously reviewed and approved by the institutional animal care and use committee (IACUC) at the Cancer Hospital of Chinese Academy of Medical Science.

### IHC staining

Sections of paraffin-embedded xenograft tumor tissue were used for IHC as we have previously described [17]. Briefly, sections were incubated with primary antibody, PARP-1, Ki67, Survivin or control IgG (1 μg/mL). After a PBS wash, they were incubated with a biotin-labeled secondary antibody. Signals were visualized using an ultrasensitive streptavidin-peroxidase system (Maxim Biotech, Fuzhou, China).

### Statistical analysis

Values are reported as the mean ± SD, and statistical significance was assessed with Student's *t* tests using Prism (v. 5, GraphPad). Tumor volume and body weight were compared between the YM155-treated and untreated groups by Student's *t* tests in the mouse xenograft assay. A *P* value of less than 0.05 was considered statistically significant.

## SUPPLEMENTARY FIGURES AND TABLES


