# Supplementary material for: YM155, a survivin suppressant, triggers PARP-dependent cell death (parthanatos) and inhibits esophageal squamous-cell carcinoma xenografts in mice
Source: Oncotarget. 2015 Jun 15;6(21):18445–59. doi: 10.18632/oncotarget.4315 (PMC4621902; doi:10.18632/oncotarget.4315)
Supplement: Supplementary file 1 [file oncotarget-06-18445-s001.pdf]

SUPPLEMENTARY FIGURES AND TABLES

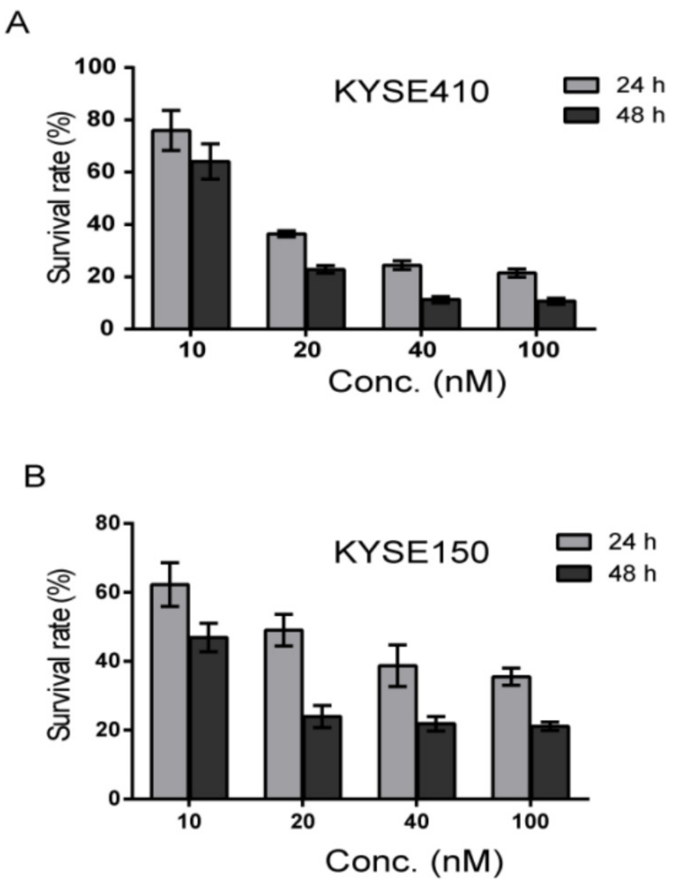

Supplementary Figure S1: Time-dependent curve for KYSE410 and KYSE150 cells after YM155 treatment for 24 and 48 h. Cells were treated with the indicated concentrations of YM155 for 24 h. The survival curves of KYSE 410 **A.** and KYSE150 **B.** cells were constructed using the CCK-8 assay.

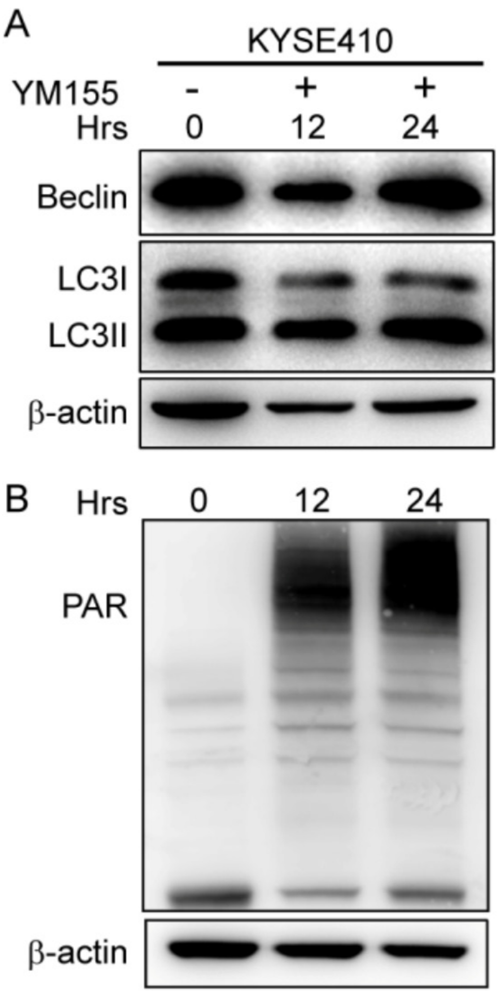

Supplementary Figure S2: A. KYSE410 cells were treated with 20 nM YM155 for 12 or 24 h. Total cell lysates were assayed for Beclin and LC3 protein expression by western blotting. Beta-actin was used as a loading control. **B.** Total KYSE410 cell protein extract was analyzed by western blotting for poly-ADP (PAR). Beta-actin was used as a loading control.

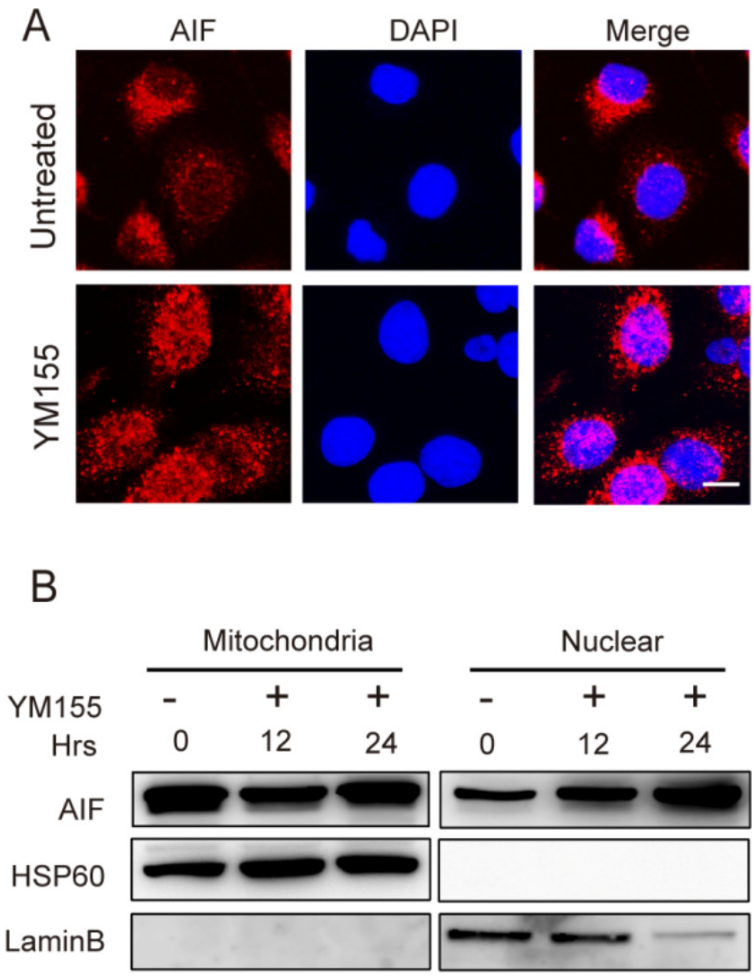

Supplementary Figure S3: YM155 induces AIF translocation. A. Nuclear accumulation of AIF in KYSE410 cells after 12 h of YM155 treatment was evaluated using immunofluorescent analysis. Nuclei were stained with DAPI, as shown in blue. Scale bars: 10 μm. **B.** Following treatment with YM155 for 12 h, cytosolic fractions were isolated from the treated cells and analyzed for AIF by western blotting. HSP60 and Lamin B protein were used as loading and fraction controls, respectively.

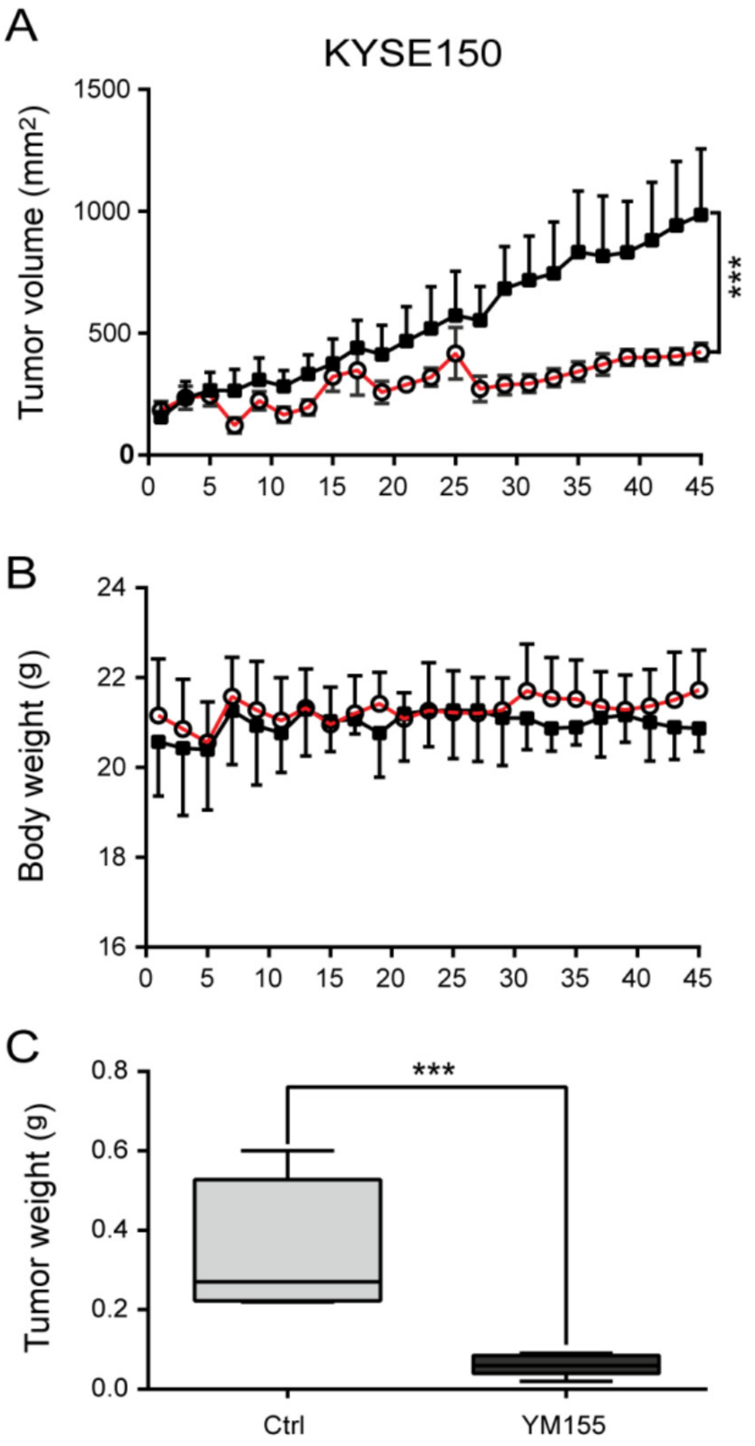

Supplementary Figure S4: Inhibition of KYSE150 tumor growth *in vivo*. A. Tumor volume was measured every 2 to 3 days after YM155 treatment. KYSE150 cells decreased the growth of the xenograft tumors compared to the untreated group ($P < 0.001$). **B.** Mouse body weight was measured over the course of 45 days. YM155 did not affect mouse body weight. **C.** Tumor weight was significantly reduced at study termination ($P < 0.05$).

Supplementary Table S1. Canonical biological process of genes up-regulated in KYSE410 by the treatment with YM155

Symbol	Genes
Regulation of programmed cell death	BAG1, CD44, CITED2, NAIP, SH3RF1, SOX9, ADRB2, ALDH1A3, CLCF1, F3, CYCS, DUSP1, GCLC, GCLM, GAS1, HSP90B1, HMOX1, ING3, IL6R, JUN, LYST, MCL1, NRG1, PTGS2, SERPINB2, BCLAF1, SPHK1, SFN, SOCS3, THBS1, TRIO, TNFAIP3, ETS1, MYC
Positive regulation of cellular biosynthetic process	CITED2, DDX5, KLF6, SERTAD1, SOX9, ADRB2, ATXN7, CREB5, EPC1, FOXA1, GRHL3, HES1, HMOX1, IL11, JUN, NFATC2, NR4A3, NFYA, PTHLH, THBS1, ETS1, MYC, ZEB1
Negative regulation of gene expression	CITED2, FOSB, LANCL2, SOX9, EPC1, EIF2C2, FST, FOXA1, GCLC, HES1, NRG1, PPARD, PURB, SIK1, BCLAF1, SNAI2, MYC, SKI, ZEB1
Negative regulation of transport	ADRB2, CALM3, FST, HMOX1, IL11, NRG1, PTGS2, THBS1
Regulation of phosphorylation	SERTAD1, ADRB2, ATXN7, CLCF1, CDC25A, CCND1, CCNE2, IL11, IL6R, JUN, NRG1, SPHK1, SFN, SOCS3, THBS1
Positive regulation of cell communication	CITED2, LANCL2, ADRB2, CLCF1, F3, GAS1, HMOX1, IL11, IL6R, PPARD, PTGS2, THBS1
Programmed cell death	BAG1, NAIP, ATXN7, F3, CYCS, GAS1, HSPB8, HMOX1, JUN, MCL1, PPARD, PHLDA2, RHOB, SGK1, SFN, THBS1, TRIO, TNFRSF21, TNFAIP3, MYC
Response to organic substance	ABCC5, BAIAP2, CD44, DNAJA1, EDEM3, CCND1, CYP1B1, DUSP1, EIF2C2, GCLC, HMOX1, IL6R, JUN, MCL1, NR4A3, PTGS2, SOCS3, THBS1, MYC
Molting cycle process	SOX9, FST, PPARD, PTGS2
Regulation of cell motion	CITED2, ARHGAP5, F3, HMOX1, IL6R, SPHK1, THBS1, ETS1
RNA processing	DDX5, DGCR14, PCF11, EIF2C2, PRPF4B, SFPQ, SFRS2B, SFRS6, SFRS7
Mitochondrion organization	NDUFAF4, JUN, SFN, SYNJ2, MYC

Up-regulated genes: induced by YM155 treatment. Underlined genes are those validated by Real-Time RT-PCR.

Supplementary Table S2. Description of primers selected for validation by Real-Time RT-PCR

Gene		Primer sequences 5′-3′	Product size(bp)
APAF1	Sence<br>Antisence	GTCACCATACATGGAATGGCA<br>CTGATCCAACCGTGTGCAAA	177
BIRC5	Sence<br>Antisence	AGGACCACCGCATCTCTACAT<br>AAGTCTGGCTCGTTCTCAGTG	118
CASP9	Sence<br>Antisence	CTGTCTACGGCACAGATGGAT<br>GGGACTCGTCTTCAGGGGAA	177
CCND1	Sence<br>Antisence	GCTGCGZZGTGGAAACCAYC<br>CCTCCTTCTGCACACCTTTGAA	135
CCNE2	Sence<br>Antisence	TCAAGACGAAGTAGCCGTTTAC<br>TGACATCCTGGGTAGTTTTCCTC	115
CYCS	Sence<br>Antisence	CTTTGGGCGGAAGACAGGTC<br>TTATTGGCGGCTGTGTAAGAG	54
FOS	Sence<br>Antisence	CACTCCAAGCGGAGACAGAC<br>AGGTCATCAGGGATCTTGCAG	139
HSPA1A	Sence<br>Antisence	GCGAGGCGGACAAGAAGAA<br>GATGGGGTTACACACCTGCT	132
JUN	Sence<br>Antisence	AACAGGTGGCACAGCTTAAAC<br>CAACTGCTGCGTTAGCATGAG	77
MLKL	Sence<br>Antisence	AGGAGGCTAATGGGGAGATAGA<br>TGGCTTGCTGTTAGAAACCTG	70
MYC	Sence<br>Antisence	GGCTCCTGGCAAAAGGTCA<br>CTGCGTAGTTGTGCTGATGT	119
NOXA	Sence<br>Antisence	ACCAAGCCGGATTTGCGATT<br>ACTTGCACTTGTTCCTCGTGG	121
SLC30A1	Sence<br>Antisence	GGACAACTTAACATGCGTGGA<br>ACACAAAAATCCCCTTCAGAACA	128
GAPDH	Sence<br>Antisence	CTGGGCTACACTGAGCACC<br>AAGTGGTCGTTGAGGGCAATG	101
